# EFNA3 Is a Prognostic Biomarker Correlated With Immune Cell Infiltration and Immune Checkpoints in Gastric Cancer

**DOI:** 10.3389/fgene.2021.796592

**Published:** 2022-01-19

**Authors:** Peng Zheng, XiaoLong Liu, Haiyuan Li, Lei Gao, Yang Yu, Na Wang, Hao Chen

**Affiliations:** ^1^ The Second Clinical Medical College of Lanzhou University, Lanzhou, China; ^2^ Abdominal Department III, Gansu Provincial Tumor Hospital, Lanzhou, China

**Keywords:** gastric cancer, EFNA3, prognosis biomarkers, immune infiltrates, immune checkpoints

## Abstract

**Background:** Ephrin A3 (EFNA3), like most genes in the ephrin family, plays a central role in embryonic development and can be dysregulated in a variety of tumors. However, the relationship between EFNA3 and gastric cancer (GC) prognosis and tumor-infiltrating lymphocytes remains unclear.

**Methods:** Tumor Immune Estimation Resource (TIMER) and Gene Expression Profiling Interactive Analysis 2 (GEPIA2) were used to analyze the expression of EFNA3. Kaplan-Meier plots and GEPIA2 were used to evaluate the relationship between EFNA3 expression and GC prognosis. Univariable survival and multivariate Cox analyses were used to compare various clinical characteristics with survival. LinkedOmics database was used for gene set enrichment analysis (GSEA). TIMER database and CIBERSORT algorithm were used to examine the relationship between EFNA3 expression and immune infiltration in GC and to explore cumulative survival in GC. The relationship between EFNA3 and immune checkpoints was examined using cBioPortal genomics analysis. Finally, EFNA3 expression in GC cells and tissues was assayed using quantitative real-time polymerase chain reaction.

**Results:** EFNA3 expression differs in a variety of cancers, and EFNA3 expression was higher in GC tissue than normal gastric tissue. GC patients with high expression of EFNA3 had worse overall survival, disease-free survival, and first progression. Multivariate analysis identified EFNA3 as an independent prognostic factor for GC. GSEA identified ribosome, cell cycle, ribosome biogenesis in eukaryotes, and aminoacyl-tRNA biosynthesis pathways as differentially enriched in patients with high EFNA3 expression. B cells, CD8^+^ T cells, CD4^+^ T cells, macrophages, neutrophils, and dendritic cells were significantly negatively correlated with a variety of immune markers. EFNA3 participates in changes in GC immune checkpoint markers in a collinear manner. EFNA3 expression in HGC-27, AGS, MKN45, and NCI-N87 was cell lines higher than that in GES-1, and patients with high expression of EFNA3 had a worse prognosis.

**Conclusion:** EFNA3 can be used as a prognostic and immune infiltration and checkpoint marker in GC patients.

## Introduction

Gastric cancer (GC) is one of the most common cancers worldwide, and the mortality rate ranks third among all cancers (Smyth et al., 2020). Surgery is the only cure for GC, but even if tumors are surgically removed, recurrence is common. Radiotherapy, chemotherapy, targeted therapy, and immunotherapy for GC are advancing rapidly, but the prognosis of patients with advanced GC remains poor. Therefore, it is very important to identify effective early diagnostic and prognostic biomarkers.

Ephrin is a general term for a class of cell surface ligands. Ephrin binds to members of the Eph tyrosine kinase receptor family and thus plays an essential role in the migration, rejection, and adhesion of neurons, blood vessels, and epithelial cells during development (Nievergall et al., 2012). Eph receptors and ephrins are signaling molecules involved in axon guidance. Recent studies have shown that they play a critical role in cancer proliferation, invasion, metastasis, and angiogenesis (Chen, 2012). Therefore, many members of the ephrin family are abnormally expressed in cancer cells, and changes in ephrin genes are often associated with greater likelihood of invasion and metastasis and worse prognosis (Kou and Kandpal, 20182018). We found that the tumor microenvironment (TME) promotes tumor growth and suppresses anti-tumor immunity *via* complex signaling pathways. Ephrins expressed in the TME play roles in tumor invasion, metastasis, and angiogenesis (Janes et al., 2021). Modulating the expression of ephrins may affect the TME and ultimately the tumor itself. In the past decade, tremendous advances in immune-related treatments and technologies have occurred. Considerable progress has been made in the development of both treatment methods and treatment techniques (Pulendran and Davis, 2020), particularly those related to immune checkpoints. Considerable research has also focused on the relationship between ephrins and immunity. Ephrin expression has been detected on both human B cells and T cells (Alonso-C et al., 2009; Luo et al., 2016), suggesting that these proteins are involved in immunity.

Our current research primarily focuses on the relationship between the expression of ephrin family proteins and various malignant tumors. For example, ephrin-A1 is highly expressed in hepatocellular carcinoma and associated with poor prognosis (Wada et al., 2014). We also found the same relationship in GC and colorectal cancer (Yuan et al., 2009; Yamamoto et al., 2013). The expression of ephrin-B1 is higher in bladder cancer tissues than normal urothelial tissue, suggesting that ephrin-B1 can be used as a biomarker of bladder cancer aggressiveness (Mencucci et al., 2020). Ephrin-B2 is also highly expressed in endometrial cancer, and patients with low ephrin-B2 expression have a better prognosis (Alam et al., 2007). As a member of the Ephrin family, EFNA3 also plays an important role in the occurrence and development of tumors. EFNA3 promotes the occurrence and development of oral tumors as well as the formation of blood vessels in oral cancer (Wang et al., 2020). EFNA3 also inhibits the proliferation and invasion of Malignant peripheral nerve sheath tumor(15). Sheath tumor cells (Wang et al., 2015). The different roles of EFNA3 in different tumors suggests the protein has diverse functions. To the best of our knowledge, only two studies examining EFNA3 in relation to GC have been published, but these studies did not examine the relationship between expression level and prognosis (Yu et al., 2020; Pei et al., 2021). In view of the role of EFNA3 in other tumors, the relationship between EFNA3 and GC requires further study.

Based on the involvement of ephrins such as EFNA3 in a variety of tumors, we hypothesized that EFNA3 would be a useful diagnostic and prognostic marker in GC patients. Although ephrins play a role in anti-cancer immunity, very little research has focused on this relationship. We therefore examined the relationship between EFNA3 expression and the immune microenvironment and immune checkpoints due to the potential usefulness of monitoring EFNA3 in clinical treatment.

In this study, we used the online tools Tumor Immune Estimation Resource (TIMER) and Gene Expression Profiling Interactive Analysis 2 (GEPIA2) to analyze the expression of EFNA3 in GC tissues. Kaplan-Meier plots and GEPIA2 were employed to explore the relationship between EFNA3 expression and GC prognosis as well as the relationship between EFNA3 and immune cell infiltration and immune checkpoints. To examine the relationship between immune checkpoints and EFNA3, gene set enrichment analysis (GSEA) was used to identify pathways enriched in GC patients with high or low expression of EFNA3. The expression of EFNA3 in GC cells and tissues as it relates to prognosis was evaluated using quantitative real-time polymerase chain reaction (qRT-PCR). The results of our research indicate that EFNA3 plays an important role in GC and clarify the relationship between EFNA3 and GC immunity.

## Materials and Methods

### Tissue Samples

A total of 50 cancerous and paracancerous tissue samples were collected from GC patients during surgery in Gansu provincial Tumor Hospital, and the tissues were stored at −80°C until analysis. Prior to analysis, tissues were homogenized, and total RNA was extracted for qRT-PCR. The study was approved by our institutional Clinical Research Ethics Committee.

### qRT-PCR

Cells were collected using a cell scraper and washed twice with cold phosphate-buffered saline. The cells were then lysed with TRIzol RNA extraction reagent (Invitrogen, Carlsbad, CA, USA) according to the manufacturer’s protocol. RNA was reversely transcribed into cDNA using the RevertAid First Strand cDNA Synthesis Kit (Thermo-Fisher Scientific, Waltham, MA, USA). Subsequently, with cDNA as the template, SYBR Premix Ex Taq™ (TaKaRa, Otsu, Shiga, Japan) was utilized for qRT-PCR. SYBR Green qPCR was used to evaluate the mRNA levels of indicated genes. Expression of target genes was normalized to that of GAPDH, and the data were analyzed according to the 2^−ΔΔCT^ method. Primers used for qRT-PCR were as follows: GAPDH, 5′-AGA​AGG​CTG​GGG​CTC​ATT​TG-3′ (F), 5′AGG​GGC​CAT​CCA​CAG​TCT​TC-3′ (R); EFNA3, TAC​TAC​TAC​ATC​TCC​ACG​CCC​ACT​C-3′ (F), 5′-TCCCGCTGATGCTCTTCTCAA-3′(R) (Zhu et al., 2021). Based on the results of qPCR, we sorted the expression levels of EFNA3 in all patients from high to low. Those with higher than the median value were the high-expression groups, and those below the median were the low-expression groups.

### Cell Culture

GES-1 gastric epithelial cells and the GC cell lines HGC-27, AGS, MKN45, and NCI-N87 were purchased from the Cell Resource Center, Peking Union Medical CollegePMUC (Beijing, China). All cells were maintained in Dulbecco’s modified Eagle’s medium (RPMI-1640; Gibco, USA) supplemented with 10% fetal bovine serum (Gibco) and 1% penicillin and streptomycin (Gibco). All cells were cultured in a 5% CO^2^ humidified atmosphere at 37°C (Baust et al., 2017).

### GEPIA2 Database Analysis

GEPIA2 (http://gepia2.cancer-pku.cn/) is a newly developed bioinformatics platform for the analysis and processing of transcriptome data from The Cancer Genome Atlas (TCGA) and Genotype-Tissue Expression (GTEx)databases.

### Survival Analysis and Prognosis Evaluation

Kaplan–Meier plots (http://kmplot.com/analysis/) were generated for prognostic analysis. Based on the median expression of EFNA3, patient samples were divided into two groups for analysis with respect to overall survival (OS), fast progression (FP), and post-progression survival (PPS). The GEPIA2 database was used to determine the prognostic value of EFNA3 expression in relation to the OS and disease-free survival (DFS) of GC patients.

### TIMER Database Analysis

TIMER (https://cistrome.shinyapps.io/timer/) is a web server for the comprehensive analysis of tumor-infiltrating immune cells and comprehensive analysis of tumor immunity. We verified the differential expression of EFNA3 between GC samples and samples of adjacent tissues (Ma et al., 2020). We also used the database to analyze EFNA3 expression in Stomach adenocarcinoma (STAD) and the correlation between EFNA3 expression and the abundance of immune infiltrating cells, including B cells, CD4^+^ T cells, CD8^+^ T cells, neutrophils, macrophages, and dendritic cells.

### Gauging the Immune Response of 22 Tumor-Infiltrating Immune Cells in GC

CIBERSORT (Gentles et al., 2015) (http://cibersort.stanford.edu/) is a deconvolution algorithm based on gene expression that can be used to evaluate changes in the expression of a set of genes relative to all other genes in the sample. We used the CIBERSORT algorithm to examine the responses of 22 TIICs(B cells naïve,B cells memory,Plasma cells, T cells CD8,T cells CD4 naïve,T cells CD4 memory resting,T cells CD4 memory activated,T cells follicular helper,T cells regulatory(Tregs),T cells gamma delta,NK cells resting,NK cells activated, Monocytes,Macrophages M0, Macrophages M1, Macrophages M2,Dendritic cells resting, Dendritic cells activated, Mast cells resting, Mast cells activated, Eosiniphils and Neutrophils) in GC in order to assess the correlations with survival and molecular subgroups.

### cBioPortal Analysis

cBioPortal (http://cbioportal.org) provides web resources for exploring, visualizing, and analyzing multi-dimensional cancer genome data (Gao et al., 2013). We used cBioPortal to visualize and compare genetic changes in the following immune checkpoint molecules: PD-L1 (CD274), PD-L2 (PDCD1LG2), CD80, CD86, VTCN1, VSIR, HHLA2, TNFRSF14, PVR, CD112 (NECTIN2), CD200, LGALS9, ICOSLG, TNFSF9, TNFSF4, CD70, TNFSF18, and CD48.

### LinkedOmics Database Analysis of EFNA3-Related Pathways

The LinkedOmics (http://www.linkedomics.org) database includes 32 cancer types from TCGA project and a total of 11,158 patients with multiple omics and clinical data. It is also the first multi-omics database that integrates mass spectrometry–based global proteomics data generated by the Clinical Proteomics Cancer Analysis Alliance on selected TCGA tumor samples. We use the LinkedOmics database to view these pathways. We also conducted GSEA in LinkInterpreter and KEGG Pathways Enrichment Analysis of EFNA3-related pathways.

### Univariate and Multivariate Cox Regression Analyses

Univariate and multivariate Cox regression were used to analyze survival. Multivariate Cox analysis was used to compare the effects of EFNA3 expression and other clinical characteristics on survival. Patients were divided into high– and low–EFNA3 expression groups. The statistical significance level for the two-tailed test was set to 0.05.

### Statistical Analysis

Survival curves were generated using GEPIA2 and Kaplan-Meier plots. The results are displayed with hazard ratio (HR) and P or Cox *p*-values from log-rank tests.

## Results

### mRNA Expression Levels of EFNA3 in Different Types of Human Cancers

**FIGURE 1:**
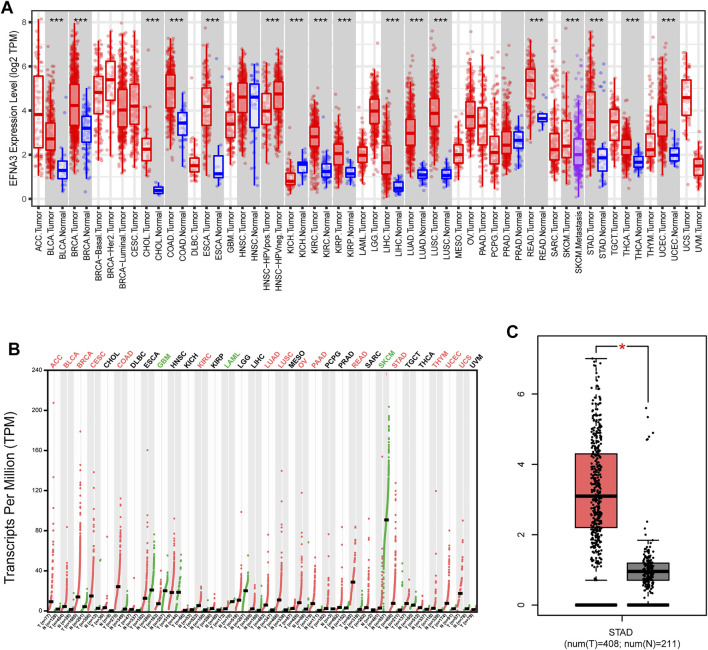
EFNA3 expression levels in different types of human cancers. **(A)** Expression of EFNA3 in different tumor types from TIMER. **(B)** Expression of EFNA3 in different tumor types in GEPIA2. **(C)** GEPIA2 generates box plots for comparing EFNA3 expression in GC and paired normal tissues (TCGA tumor versus TCGA normal + GTEx normal). (*P< 0.05, **P< 0.01, ***P< 0.001).

To evaluate differences in EFNA3 expression in tumor and normal tissues, the EFNA3 mRNA levels in tumor and normal tissues of patients with multiple types of cancer were analyzed using the TIMER database. EFNA3 expression was higher in BLCA, CHOL, COAD, KIRC, ESCA, NISC, KIRC, KIRP, LIHC, LUAD, LUSC, READ, SKCM, STAD, THCA, and UCEC compared with normal tissues. In addition, lower expression was observed in KICH (Figure 1A). In GEPIA2, we observed high expression in ACC, BLCA, RBCA, COAD, KIRC, LUAD, LUSC, OV, PAAD, READ, STAD, THYM, UCEC, and UCS and low expression in GBM, LAML, and SKCM (Figure 1B). EFNA3 was highly expressed in STAD (Figure 1C).

### Prognostic Utility of EFNA3 in GC

**FIGURE 2:**
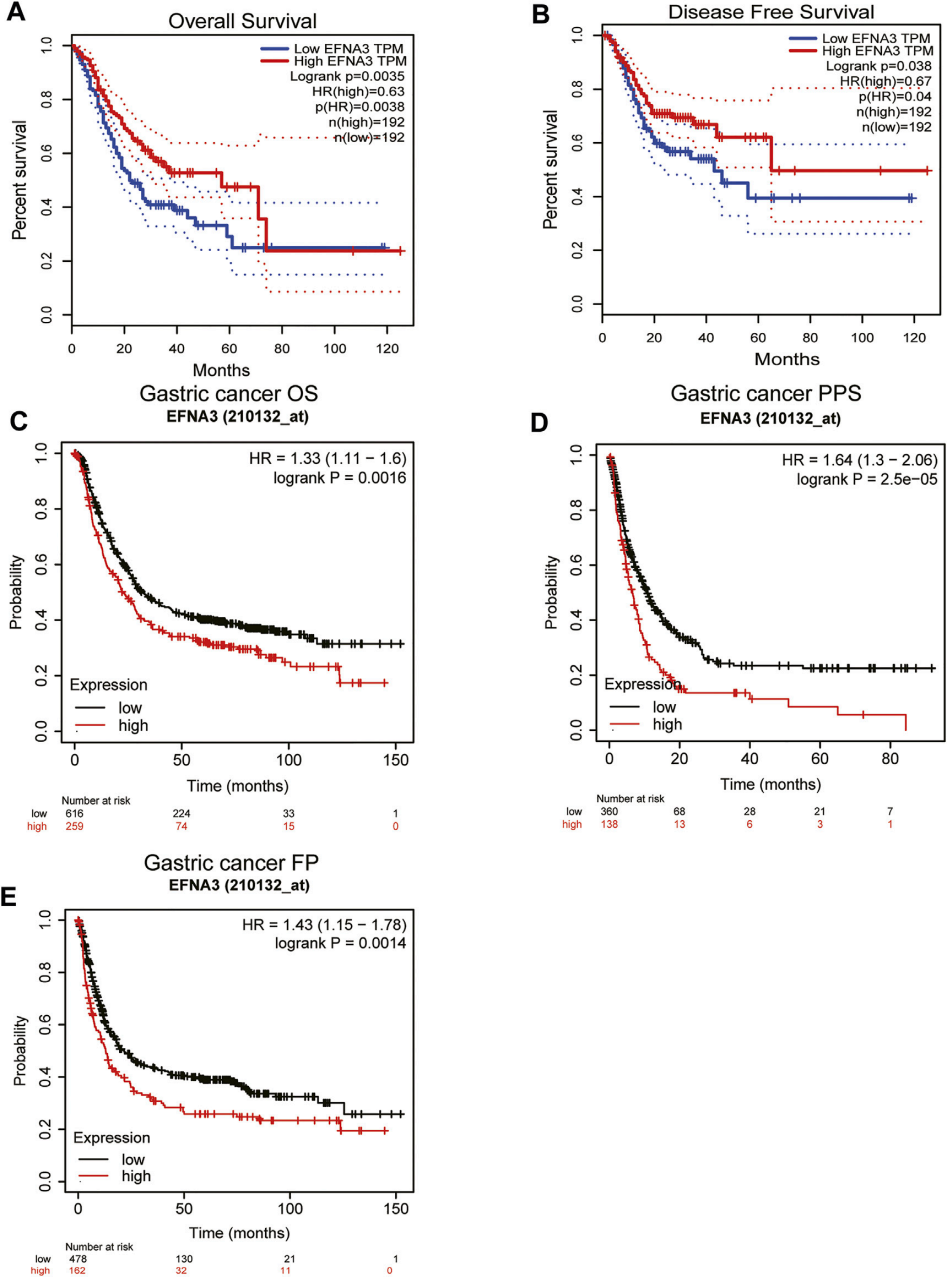
Correlation of EFNA3 expression with prognostic value in GC. Survival curves of differential EFNA3 expression were analyzed using GEPIA2 **(A, B)**. Correlation between EFNA3 and prognosis of STAD in the Kaplan-Meier plot database **(C**–**E)**. OS, overall survival; DFS, disease-free survival; FP, fast progression; PPS, post-progression survival.

The prognostic value of EFNA3 expression in GC was evaluated using Kaplan-Meier plots and GEPIA2. Expression of EFNA3 was significantly associated with the prognosis of GC patients. The results of GEPIA2 analysis showed that high expression of EFNA3 was associated with longer OS (HR = 0.63, *p* = 0.0038) and DFS (HR = 0.67, *p* = 0.04) of GC patients (Figures 2A,B) compared with GC patients with low EFNA3 expression. In analyses using the KM web tool, the OS (HR = 1.33 [1.11–1.6], *p* = 0.0016), PPS (HR = 1.64 [1.3–2.06], *p* = 2.5e-05), and FP (HR = 1.43 [1.15–1.78], *p* = 0.0014) of GC patients with high EFNA3 expression (Figures 2C–E) values were significantly lower than those of patients with low EFNA3 expression.

In order to better understand the relationship between the expression of EFNA3 and GC, we examined the expression of EFNA3 in relation to various clinical characteristics in GC patients using the KM web tool. High expression of EFNA3 in males and females with stage 3 disease of intestinal type was associated with poor OS, PPS, and FP. In terms of differentiation, high expression of EFNA3 was associated with poor OS, regardless of high, medium, or low differentiation (Table 1). Finally, we downloaded GC-related information from TCGA, remove the missing information. Univariate and multivariate Cox analysis identified EFNA3 expression (HR = 0.701 [0.504–0.974], *p* = 0.034) as an independent prognostic factor in patients with GC, (Table 2).

**TABLE 1:** Correlation of EFNA3 mRNA expression and clinical prognosis in gastric cancer with different clinicopathological factors by Kaplan-Meier plotter Clinicopathological characteristics. Bold values indicate *p* < 0.05.

	Overall survival (*n* = 875)			First progression (*n* = 640)		
	N	Hazard ratio	*p*-value	N	Hazard ratio	*p*-value
SEX	—	—	—	—	—	—
—	236	1.58 (1.09–2.28)	**0.015**	201	1.58 (1.05–2.37)	**0.025**
—	534	1.35 (1.09–1.67)	**0.006**	437	1.36 (1.06–1.76)	**0.017**
STAGE	—	—	—	—	—	—
1	67	0.63 (0.23–1.74)	0.37	60	0.63 (0.19–2.06)	0.44
2	140	0.58 (0.3–1.1)	0.091	131	1.69 (0.78–3.66)	0.18
3	305	1.5 (1.09–2.08)	**0.013**	186	1.99 (1.28–3.08)	**0.002**
4	148	0.68 (0.46–1)	**0.049**	141	0.62 (0.41–0.94)	**0.024**
STAGE T	—	—	—	—	—	—
2	241	0.73 (0.47–1.14)	0.16	239	0.71 (0.42–1.17)	0.17
3	204	1.52 (1.07–2.16)	**0.019**	204	1.35 (0.96–1.89	0.085
4	38	0.44 (0.19–1.02)	0.05	39	0.52 (0.22–1.21)	0.12
STAGE N	—	—	—	—	—	—
0	74	0.54 (0.23–1.26)	0.15	72	0.6 (0.26–1.39)	0.23
1	225	1.98 (1.18–3.31	**0.009**	222	1.98 (1.18–3.27)	**0.008**
2	121	2.25 (1.14–3.56)	**0.001**	125	1.79 (1.15–2.78)	**0.009**
3	76	0.62 (0.36–1.07)	0.083	76	0.61 (0.35–1.04)	0.068
1 + 2 + 3	442	1.26 (0.96–1.65)	0.096	423	1.29 (0.97–1.72)	0.084
STAGE M	—	—	—	—	—	—
0	444	1.29 (0.97–1.71)	0.08	443	1.25 (0.95–1.65)	0.11
1	241	1.83 (0.98–3.4)	0.054	56	0.7 (0.38–1.31)	0.26
LAUREN CLASSIFICATION	—	—	—	—	—	—
Intestinal	320	1.67 (1.22–2.3)	**0.001**	263	1.53 (1.08–2.18)	**0.017**
Diffuse	241	1.38 (0.97–1.98)	0.075	231	1.39 (0.97–1.99)	0.068
Mixed	32	0.42 (0.15–1.19)	0.095	28	0.2 (0.06–0.69)	0.0057
DIFFERENTIATION	—	—	—	—	—	—
Poor	165	0.58 (0.38–0.88)	**0.01**	121	0.59 (0.37–0.96)	**0.03**
Moderate	67	2.5 (1.17–5.35)	**0.014**	67	2.27 (1.1–4.68)	**0.023**
Well	32	0.35 (0.13–0.97)	**0.034**	—	—	—
—	First Progression (n = 640)	—	—	—	—	—
—	N	Hazard ratio	*p*-value	—	—	—
SEX	—	—	—	—	—	—
—	149	1.63 (1.04–2.53))	**0.03**	—	—	—
—	348	1.67 (1.28–2.17)	**0.001**	—	—	—
STAGE	—	—	—	—	—	—
2	105	0.56 (0.28–1.09)	**0.001**	—	—	—
3	142	2.77 (1.79–4.27)	**0.002**	—	—	—
4	104	0.69 (0.43–1.08)	0.1	—	—	—
STAGE T	—	—	—	—	—	—
2	196	0.71 (0.42–1.17)	0.17	—	—	—
3	150	1.35 (0.96–1.89	0.085	—	—	—
4	—	—	—	—	—	—
STAGE N	—	—	—	—	—	—
1	169	1.89 (1.11–3.21)	**0.017**	—	—	—
2	105	2.64 (1.62–4.32)	**0.009**	—	—	—
3	63	0.57 (0.31–1.06)	**0.001**	—	—	—
1 + 2 + 3	337	1.44 (1.07–1.94)	**0.014**	—	—	—
STAGE M	—	—	—	—	—	—
0	342	1.47 (1.08–2)	**0.013**	—	—	—
LAUREN CLASSIFICATION	—	—	—	—	—	—
Intestinal	192	2.39 (1.56–3.66)	**0.001**	—	—	—
Diffuse	176	1.34 (0.91–1.98)	0.14	—	—	—

**TABLE 2:** Univariate COX regression analysis and Multivariate COX regression analysis for EFNA3.

Characteristics	Total(N)	Univariate analysis		Multivariate analysis	
		Hazard ratio (95% CI)	*p* Value	Hazard ratio (95% CI)	*p* Value
Age (>65 *vs*. ≤65)	367	1.620 (1.154–2.276)	**0.005**	1.974 (1.364–2.857)	**<0.001**
Gender (Male *vs*. Female)	370	1.267 (0.891–1.804)	0.188	—	—
T stage (T3&T4 *vs*. T1&T2)	362	1.719 (1.131–2.612)	**0.011**	1.446 (0.919–2.276)	**0.111**
N stage (N2&N3 *vs*. N0&N1)	352	1.650 (1.182–2.302)	**0.003**	1.542 (1.086–2.190)	**0.015**
M stage (M1 *vs*. M0)	352	2.254 (1.295–3.924)	**0.004**	2.860 (1.593–5.133)	**<0.001**
EFNA3 (High *vs*. Low)	370	0.703 (0.506–0.978)	**0.036**	0.625 (0.439–0.889)	**0.009**

### Identification of EFNA3-Related Signaling Pathways Using GSEA

**FIGURE 3:**
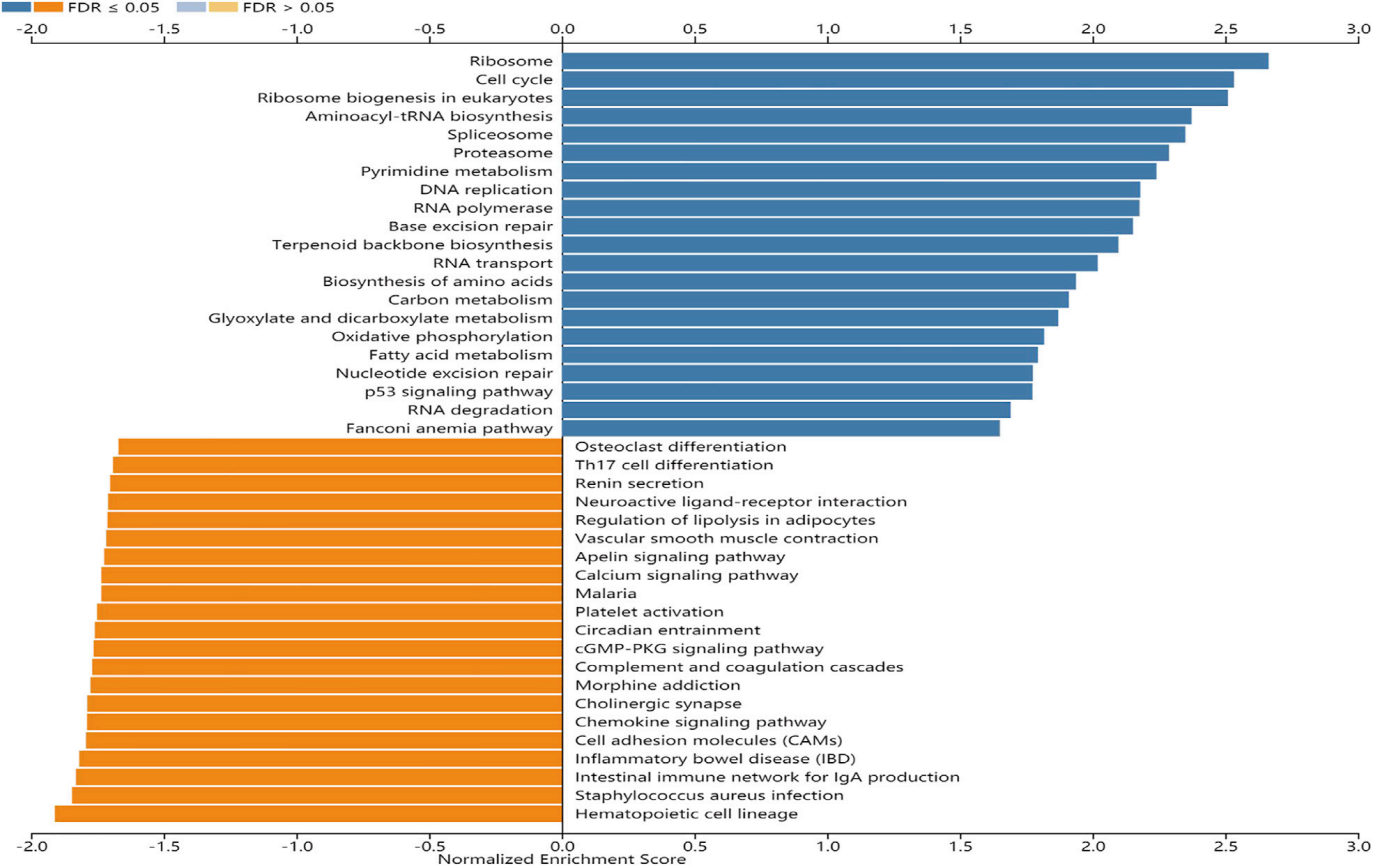
Enrichment plots from GSEA. KEGG pathways of EFNA3 in STAD cohort. FDR, false-discovery rate.

GSEA was performed to identify signaling pathways that are activated in GC. Ribosome, cell cycle, ribosome biogenesis in eukaryotes, and aminoacyl-tRNA biosynthesis pathways were differentially enriched and positively correlated with EFNA3 mRNA expression phenotype. In contrast, hematopoietic cell lineage, *Staphylococcus aureus* infection, intestinal immune network for IgA production, and inflammatory bowel disease pathways were negatively correlated with EFNA3 mRNA expression (Figure 3).

### Relationship Between EFNA3 Expression and TIICs

**FIGURE 4:**
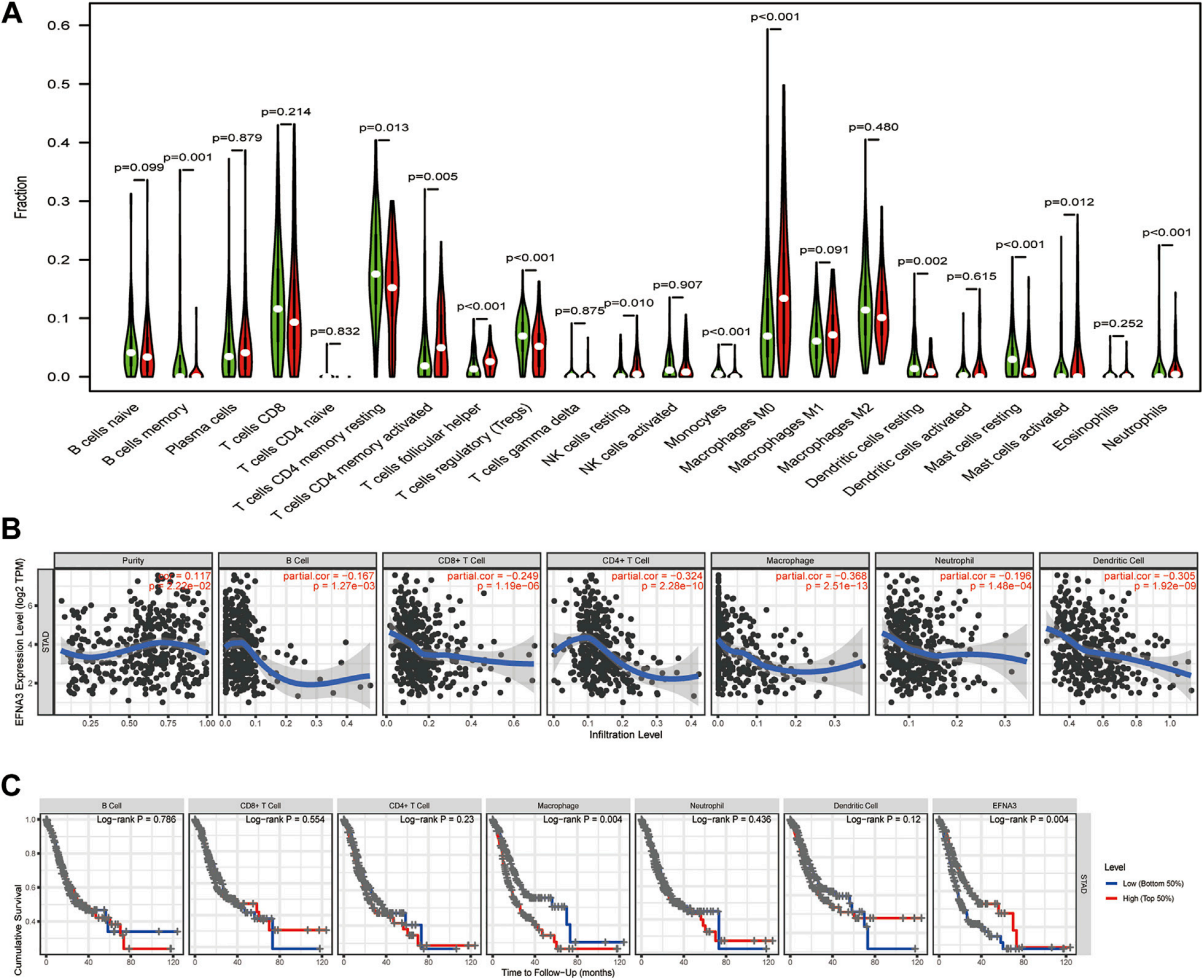
**(A)** Proportion of 22 subpopulations of immune cells. **(B)** EFNA3 expression level was significantly negatively correlated with levels of infiltrating B cells, CD8^+^ T cells, CD4^+^ T cells, macrophages, neutrophils, and DCs in GC. **(C)** Cumulative survival was related to macrophages and EFNA3 in GC.

We also evaluated whether the expression of EFNA3 is related to immune cell infiltration in GC using data downloaded from TCGA. Tumor specimens were divided into groups based on high and low EFNA3 expression. We used CIBERSORT to calculate and download the gene expression profiles of the samples to infer the immune infiltration of 22 immune cells. The results showed that memory B cells, memory resting CD4 T cells, follicular T helper cells, regulatory T cells, resting NK cells, monocytes, M0 macrophages, resting dendritic cells, resting mast cells, activated mast cells, and neutrophils were the primary immune cells affected by the expression of EFNA3 (Figure 4A).

### TIMER Analysis of Correlation Between EFNA3 Expression and Immune Cell Infiltration Level and Cumulative Survival in GC

As TIICs are independent predictors of cancer prognosis, it is very important to study the relationship between the expression of EFNA3 and the level of immune cell infiltration. Using the TIMER database, we found that EFNA3 expression was significantly negatively correlated with the infiltration of B cells, CD8^+^ T cells, CD4^+^ T cells, macrophages, neutrophils, and dendritic cells (Figure 4B). Macrophage infiltration and EFNA3 expression were related to the cumulative survival rate of GC patients over time (Figure 4C).

In order to further characterize the role of EFNA3 expression and TIICs, we analyzed the relationship between the expression of EFNA3 and immune marker genes in different types of immune cells, including CD8^+^ T cells, T cells (general), B cells, monocytes, tumor-associated macrophages (TAMs), M1 and M2 macrophages, neutrophils, NK cells, DCs, Th1 cells, Th2 cells, follicular T helper cells, Th17 cells, Tregs, and T cell exhaustion. We found that EFNA3 expression was also related to several immune markers of B cells, CD8^+^ T cells, CD4^+^ T cells, macrophages, neutrophils, and dendritic cells. These results were consistent with our previous results. Interestingly, the expression of EFNA3 was not related to M1 macrophages but closely related to M2 macrophages. In addition, the expression levels of most marker sets of monocytes and TAMs were closely related to the expression of EFNA3 (Table 3).

**TABLE 3:** Correlation analysis between EFNA3 and relate genes and markers of immune cells in TIMER.

Description	Gene markers	None			Purity
		Cor	P	Cor	P
**CD8** ^**+**^ **T cell**	—	—	—	—	—
—	CD8A	−0.207315046	***	−0.20242845	***
—	CD8B	−0.149493123	**	−0.143242331	**
**T cell (general)**	—	—	—	—	—
—	CD3D	−0.263887993	***	−0.249219653	***
—	CD3E	−0.267123175	***	−0.26059211	***
—	CD2	−0.238296665	***	−0.224033483	***
**B cell**	—	—	—	—	—
—	CD19	−0.257111288	***	−0.242635831	***
—	CD79A	−0.304089267	***	−0.295011059	***
**Monocyte**	—	—	—	—	—
—	CD86	−0.174594623	***	−0.159553219	**
—	CD115 (CSF1R)	−0.232143121	***	−0.217217322	***
**TAM**	—	—	—	—	—
—	CCL2	−0.170170145	**	-0.150541669	**
—	CD68	−0.110379646	*	-0.10019684	0.051284861
—	IL10	−0.151439718	*	−0.11591185	*
**M1 Macrophage**	—	—	—	—	—
—	INOS (NOS2)	0.01731189	0.725113784	0.033387321	0.516974952
—	IRF5	−0.058798236	0.231890806	−0.051855302	0.314003055
—	COX2(PTGS2)	0.038192905	0.437599325	0.04611645	0.370625499
**M2 Macrophage**	—	—	—	—	—
—	CD163	−0.117692005	*	−0.100753601	*
—	VSIG4	−0.128470571	**	−0.108138566	*
—	MS4A4A	−0.245048757	***	−0.228561813	***
**Neutrophils**	—	—	—	—	—
—	CD66b (CEACAM8)	−0.038892665	0.429403341	−0.045277856	0.379398546
—	CD11b (ITGAM)	−0.237196109	***	−0.222545419	***
—	CCR7	−0.328484339	***	−0.303657644	***
**Natural killer cell**	—	—	—	—	—
—	KIR2DL1	−0.054005227	0.272356068	−0.046098831	0.370808521
—	KIR2DL3	−0.074236678	0.131084612	−0.048572102	0.345664963
—	KIR2DL4	0.052175156	0.288960296	0.080487011	0.117752329
—	KIR3DL1	−0.047502648	0.33438018	−0.044153189	0.391362199
—	KIR3DL2	−0.092480122	0.059793067	−0.081231199	0.11438445
—	KIR3DL3	0.002491516	0.959641852	−0.001757506	0.972795818
—	KIR2DS4	−0.015480848	0.753189563	−0.036168244	0.482663905
**Dendritic cell**	—	—	—	—	—
—	HLA-DPB1	−0.25863437	***	−0.235387886	***
—	HLA-DQB1	−0.141898195	*	−0.111787485	*
—	HLA-DRA	−0.155704255	**	−0.124386463	*
—	HLA-DPA1	−0.188483029	**	−0.158691397	*
—	BDCA-1(CD1C)	−0.419921274	***	−0.395322677	***
—	BDCA-4(NRP1)	-0.256360056	***	-0.251782132	***
—	CD11c (ITGAX)	−0.164137654	**	−0.145959864	*
**Th1**	—	—	—	—	—
—	T-bet (TBX21)	−0.211737847	***	−0.191068168	**
—	STAT4	−0.280788203	***	−0.28766748	***
—	STAT1	0.104556017	*	0.107212799	*
—	IFN-γ (IFNG)	0.036859801	0.453928996	0.055368894	0.28229336
—	TNF-α (TNF)	−0.013194081	0.788624466	0.030997142	0.547439608
**Th2**	—	—	—	—	—
—	GATA3	−0.268551295	***	−0.27844804	***
—	STAT6	−0.1308076	*	−0.135425002	*
—	STAT5A	−0.10826853	*	−0.101518944	*
—	IL13	−0.050161416	0.30800105	−0.042617284	0.408064011
**Tfh**	—	—	—	—	—
—	BCL6	−0.057315888	0.243998933	−0.049118066	0.340263959
—	IL21	−0.006365356	0.897134245	0.018712321	0.716516372
**Th17**	—	—	—	—	—
—	STAT3	−0.061558105	0.210680659	−0.057538398	0.263832341
—	IL17A	−0.019216313	0.696298946	−0.000146833	0.997726758
**Treg**	—	—	—		—
—	FOXP3	−0.119564378	*	−0.107616351	*
—	CCR8	−0.129723551	*	−0.115791686	*
—	STAT5B	−0.204293596	***	−0.202750648	***
—	TGFβ (TGFB1)	−0.125655895	*	−0.125169215	*
**T cell exhaustion**	—	—	—	—	—
—	PD-1 (PDCD1)	−0.104825489	*	−0.084697986	0.099680031
—	CTLA4	−0.016864448	0.731835488	0.011626566	0.821508615
—	LAG3	−0.065025822	0.186062406	−0.053968224	0.294665055
—	TIM-3 (HAVCR2)	−0.126420397	*	−0.106746361	*
—	GZMB	0.096918275	*	0.122835389	*

### EFNA3 and Immune Checkpoints

**FIGURE 5:**
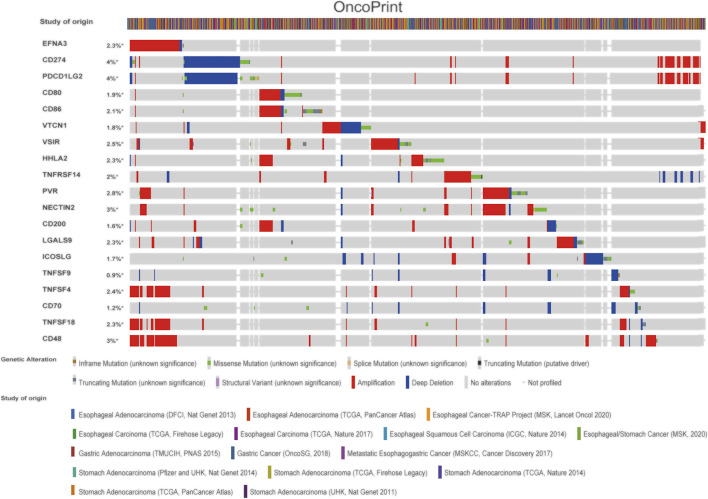
Landscape of EFNA3 and immune checkpoint changes in GC. Compact visualization of cases with multiple genetic alterations in EFNA3 and immune checkpoints (derived from 15 studies) shown individually by cBioPortal as indicated, including fusions, amplifications, deep deletions, truncating mutations, and missense mutations.

We also explored the genetic changes in the EFNA3 gene and the immune checkpoints we mentioned earlier in GC. The general landscape of EFNA3 and immune checkpoint alteration in GC was compactly visualized, including fusion, amplification, deep deletion, truncating, and missense mutations (Figure 5). Genetic alterations in EFNA3 in GC reached as high as 3%, a level higher than that of other immune checkpoint changes (Figure 5).

Next, we examined the relationship between EFNA3 and each representative immune checkpoint separately. Mutations in EFNA3 exhibited statistically significant co‐occurrences rather than mutual exclusivity with a variety of immune checkpoints, such as CD48, TNFSF4, TNFSF18, PVR, NECTIN2, CD274, and TNFRSF14 (Table 4).

**TABLE 4:** Mutual-exclusivity analysis between EFNA3 and multiple-immune checkpoints in gastric cancer.

A	B	Neither	A not B	B not A	Both	Log2 odds ratio	*p*-Value	q-Value	Tendency	Significant
EFNA3	CD48	2,374	10	34	51	>3	**<**0.001	**<**0.001	Co-occurrence	Yes
EFNA3	TNFSF4	2,384	21	24	40	>3	**<**0.001	**<**0.001	Co-occurrence	Yes
EFNA3	TNFSF18	2,384	25	24	36	>3	**<**0.001	**<**0.001	Co-occurrence	Yes
EFNA3	PVR	2,347	47	61	14	>3	**<**0.001	**<**0.001	Co-occurrence	Yes
EFNA3	NECTIN2	2,335	53	73	8	2.271	**<**0.001	0.004	Co-occurrence	Yes
EFNA3	CD274	2,290	53	118	8	1.551	0.011	0.042	Co-occurrence	Yes
EFNA3	TNFRSF14	2,346	56	62	5	1.756	0.023	0.071	Co-occurrence	Yes
EFNA3	LGALS9	2,353	57	55	4	1.586	0.056	0.145	Co-occurrence	No
EFNA3	CD200	2,373	58	35	3	1.81	0.066	0.158	Co-occurrence	No
EFNA3	VSIR	2,349	57	59	4	1.482	0.068	0.162	Co-occurrence	No
EFNA3	TNFSF9	2,385	59	23	2	1.814	0.125	0.249	Co-occurrence	No
EFNA3	CD80	2,360	59	48	2	0.737	0.352	0.503	Co-occurrence	No
EFNA3	PDCD1LG2	2,290	57	118	4	0.446	0.356	0.503	Co-occurrence	No
EFNA3	CD86	2,357	59	51	2	0.648	0.379	0.512	Co-occurrence	No
EFNA3	HHLA2	2,351	59	57	2	0.484	0.432	0.535	Co-occurrence	No
EFNA3	CD70	2,378	60	30	1	0.402	0.542	0.59	Co-occurrence	No
EFNA3	VTCN1	2,355	60	53	1	−0.433	0.612	0.642	Mutual exclusivity	No
EFNA3	ICOSLG	2,361	60	47	1	−0.256	0.666	0.674	Mutual exclusivity	No

### Elevated EFNA3 Expression in GC Cell Lines and Tissues

**FIGURE 6:**
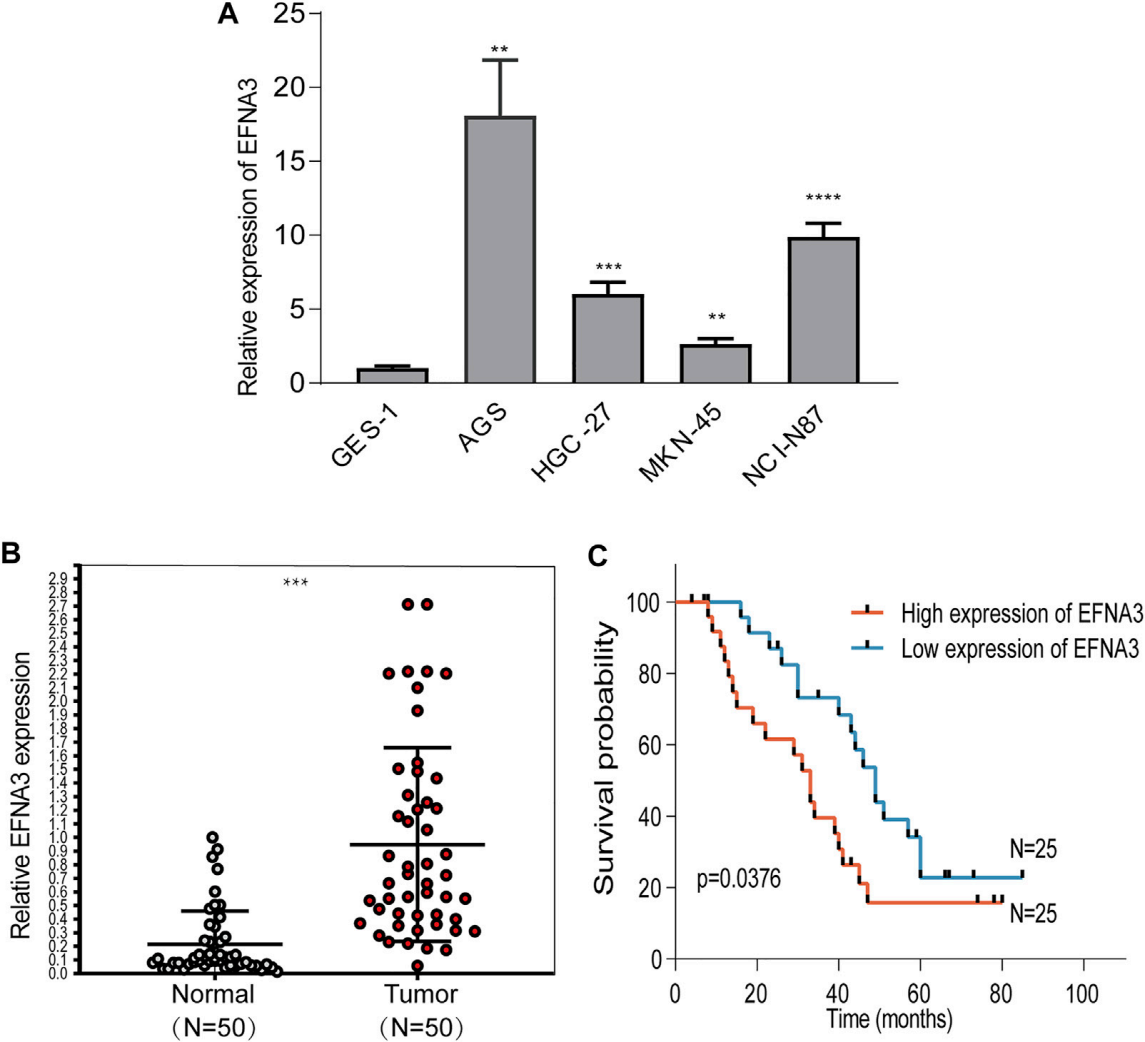
EFNA3 expression was significantly up-regulated in cell lines and GC tissues. qRT-PCR showed that EFNA3 expression was up-regulated in GC cell lines **(A)** and GC tissues **(B)**. High EFNA3 expression was related to shorter OS time **(C)**. (**P< 0.01, ***P< 0.001, ****P< 0.0001).

In order to characterize EFNA3 expression in GC tissues and cell lines, qRT-PCR was performed, and the results showed that EFNA3 expression was significantly higher in GC tissues than adjacent non-cancerous tissues (Figure 6B). In GC cell lines, the expression of EFNA3 was significantly higher than in GES-1 cells (Figure 6A). In addition, after grouping patients based on high versus low EFNA3 expression, log-rank tests showed that high EFNA3 expression was associated with poor prognosis (Figure 6C).

## Discussion

Cancer cells at different stages of transformation and metastasis rely on signal transduction between cells. Ephs/Ephrins act as repulsive and attractive signaling molecules between cells and can bind to Eph receptors on neighboring cells, resulting in contact-dependent bidirectional signal transduction between neighboring cells (Kandouz, 2012). Abnormal signal transduction leads to the occurrence and development of tumors. Our research focuses on the ephrin family member EFNA3. Although EFNA3 has not been extensively studied, available research in sheath tumor and oral cancers is gratifying (Yuan et al., 2009; Yamamoto et al., 2013). Here, we studied the expression of EFNA3 in different cancers, focusing on the high expression of EFNA3 in GC and its relationship to poor prognosis. In addition, the expression level of EFNA3 in GC is related to the levels of immune cell infiltration and different immune markers.

The online data results of our study show that the expression of EFNA3 in many types of cancers differs from that in normal tissues. EFNA3 is highly expressed in GC, hepatocellular carcinoma, and other cancers. We found that KICH expression was low in the TIMER database, but there was no difference in the GEPIA2 data. By comparison, SKCM expression was high in TIMER but low in GEIPA2. Differences in expression in the same cancer noted in different databases may be related to differences in data collection methods, statistical analyses, and biological characteristics. In both databases we examined, EFNA3 was highly expressed in GC, consistent with our qRT-PCR results. Kaplan-Meier plot analyses of OS between the databases showed that GC patients with high expression of EFNA3 had a poor prognosis, which was also closely related to gender and classification, stages 3 and 4, stage T3, stages N1 and 2, classification of intestinal, whereas the prognosis of GC patients with high expression of EFNA3 in GEPIA2 was good. The prognostic differences between databases may be related to the study subject inclusion and rejection criteria, the amount of specimens analyzed, as well as other human or random factors. Therefore, we grouped GC patients based on EFNA3 expression from the results of qRT-PCR analyses. Our results show that GC patients with high expression of EFNA3 have a significantly worse prognosis than GC patients with low EFNA3 expression (*p* = 0.0376). The prognostic utility of EFNA3 for GC patients was further evaluated using univariate and multivariate Cox analyses, which indicated that EFNA3 is a useful independent prognostic factor for GC. These results strongly indicate that EFNA3 is a promising prognostic biomarker for GC.

Based on our initial results, we sought to identify signaling pathways that are enriched in GC patients with high expression of EFNA3, because these patients are at higher risk of poor outcome. The results of GSEA showed that high expression of EFNA3 was associated primarily with enrichment of six pathways. The most markedly enriched pathway was the ribosome pathway. In cancer cells, increased ribosome synthesis leads to a corresponding increase in protein synthesis, which plays an important role in the development of most tumors. Inhibition of ribosome biosynthesis has become a new target in cancer treatment (Pelletier et al., 2018; Catez et al., 2019). The change in EFNA3 expression leads to enrichment of the ribosome pathway, indicating that EFNA3 expression is closely related to ribosome biosynthesis in GC cells. Further interactions need to be verified by related experiments; however, our present research still provides new insights regarding the treatment of GC.

Studies of immune cell infiltration have shown that immune cells in the TME play an important role in the progression of cancer (Lei et al., 2020). A deeper understanding of immune cell infiltration in the immune microenvironment could facilitate the development of new strategies for cancer immunotherapy. Our results show that the expression of EFNA3 is negatively correlated with the infiltration of a variety of immune cells, with the highest correlation with macrophages (Cor = −0.368, *p* = 2.51e-13). Based on that result, we explored tumor-associated macrophages (TAMs) and genetic markers of M1 and M2 macrophages. Interestingly, the three genetic markers of M1 macrophages were not correlated with the expression of EFNA3, whereas the expression of the three genetic markers of M2 macrophages examined were closely related to the expression of EFNA3. M1 macrophages mainly participate in positive immune responses such as immune surveillance and inhibition of tumor growth, whereas M2 macrophages mainly secrete inhibitory cytokines (such as IL-10 and TGF-β) to down-regulate the immune response, thereby promoting tumor growth (Cortese et al., 2020). Therefore, the close relationship between EFNA3 expression and M2 macrophages may be related to its M2 macrophages down-regulation of the immune response. We know that TAMs are not identical to the M1 and M2 macrophage subtypes, but TAMs are similar to M2 macrophages and promote tumor growth by inducing immunosuppression (Mehla and Singh, 2019). M2 macrophages also cooperate with Th2 and Treg cells to affect multiple steps of tumor development (Najafi et al., 2019). The expression of markers of Th2 cells (GATA3, STAT6, STAT5A) and Tregs (FOXP3, CCR8, STAT5B, TGF-β [TGFB1]) differed significantly. These results may indicate that EFNA3 has the potential to regulate TAMs. We therefore examined the relationship between high and low expression of EFNA3 and 22 types of immune cells and found that high EFNA3 expression is correlated is correlated with M2 macrophages (*p* = 0.034), consistent with the TIMER results. In our research, we found many articles related to ephrins and T cells. For example, in GC, EFNB1 inhibits T cells *via* follicular T helper cells (Lu et al., 2017). In experimental autoimmune encephalomyelitis and multiple sclerosis, the expression of EFNB1 and EFNB2 was found to be related to the migration of T cells (Luo et al., 2016). Based on this observation, we explored TIICs because the analysis of TIICs in human tumors usually focuses on T cells. We found that EFNA3 is highly expressed primarily by infiltrating activated CD4 memory T cells and follicular T helper cells, whereas low EFNA3 expression is primarily associated with infiltrating resting CD4 memory T cells and Tregs.

The blocking of immune checkpoints is increasingly considered a primary future method for cancer immunotherapy. However, at least in current clinical practice, the treatment of GC is focused primarily on surgery and radiotherapy (Zhao et al., 2019). Genomic investigations showed that EFNA3 actually participates in the changes in immune checkpoints. Changes of EFNA3 in expression co-occur with changes in a wide range of immune checkpoints (CD48, TNFSF4, TNFSF18, PVR, NECTIN2, CD274, and TNFRSF14), which strongly suggest that EFNA3 is a co-regulator of immune checkpoints in GC.

Although the present study further elucidated the relationship between EFNA3 expression and prognosis in GC through analyses involving multiple databases and experiments, the pathogenic mechanism of EFNA3 in GC was only examined to a limited degree using GSEA. Further studies are needed to verify our present results. In addition, the reasons for the prognostic differences between the different databases could not be conclusively determined. In order to eliminate potentially interfering factors, studies with larger sample sizes will be needed to minimize potential errors. Our study was limited by the small sample size and therefore could not fully elucidate the relationship between EFNA3 expression and GC prognosis. Finally, although we studied the relationship between EFNA3 expression and immune checkpoints using online databases, clearly determining this relationship requires further confirmation. Although these problems will likely be solved in the future, our research clearly shows that GC tissues express significantly higher levels of EFNA3, and high expression of EFNA3 is associated with a worse outcome in GC, as it is closely related to immune cell infiltration and regulation of immune checkpoints. In short, EFNA3 appears to hold tremendous promise as both a target in GC immunotherapy and a promising prognostic indicator of GC.

## Data Availability

The datasets presented in this study can be found in online repositories. The names of the repository/repositories and accession number(s) can be found in the article/Supplementary Material.
